# A Two-Layered Diffusion Model Traces the Dynamics of Information Processing in the Valuation-and-Choice Circuit of Decision Making 

**DOI:** 10.1155/2014/383790

**Published:** 2014-08-31

**Authors:** Pietro Piu, Francesco Fargnoli, Alessandro Innocenti, Alessandra Rufa

**Affiliations:** ^1^Department of Medicine, Surgery & Neurosciences, University of Siena, Viale Bracci 2, 53100 Siena, Italy; ^2^Eye-Tracking & Visual Application Lab, University of Siena, Viale Bracci 2, 53100 Siena, Italy; ^3^Department of Social, Political and Cognitive Sciences, University of Siena, Via Roma 56, 53100 Siena, Italy

## Abstract

A circuit of evaluation and selection of the alternatives is considered a reliable model in neurobiology. The prominent contributions of the literature to this topic are reported. In this study, valuation and choice of a decisional process during Two-Alternative Forced-Choice (TAFC) task are represented as a two-layered network of computational cells, where information accrual and processing progress in nonlinear diffusion dynamics. The evolution of the response-to-stimulus map is thus modeled by two linked diffusive modules (2LDM) representing the neuronal populations involved in the valuation-and-decision circuit of decision making. Diffusion models are naturally appropriate for describing accumulation of evidence over the time. This allows the computation of the response times (RTs) in valuation and choice, under the hypothesis of ex-Wald distribution. A nonlinear transfer function integrates the activities of the two layers. The input-output map based on the infomax principle makes the 2LDM consistent with the reinforcement learning approach. Results from simulated likelihood time series indicate that 2LDM may account for the activity-dependent modulatory component of effective connectivity between the neuronal populations. Rhythmic fluctuations of the estimate gain functions in the delta-beta bands also support the compatibility of 2LDM with the neurobiology of DM.

## 1. Introduction

Even simple decisions imply higher cognitive functions that integrate noisy sensory stimuli, prior knowledge, and the costs-and-benefits related to possible actions in function of their time of occurrence. Accumulation of noisy information is a reliable pattern performed by neural pools in cortical circuitry during decision making (DM) process. This process is time absorbing, especially when the quality of information is poor and there exist many possible alternatives that may be evaluated and compared. There exists large consensus in the studies of DM toward the conformation of a phase of accumulation of evidence until a decision is made [1–11]; that is, the decision maker is expected to keep on gathering information until the evidence in favor of one of the alternatives suffices. Thus, the stochastic integration of information up to a certain threshold gives rise to a speed-accuracy tradeoff (the performance of the responses increases for slower response times) that is bounded by the costs associated with obtaining more information. In this context the responses times (RTs) to the stimuli characterize the speed-accuracy tradeoff because they allow the identification of the time when a decision is made (although not yet completed by the motor action) [12]. RT studies have addressed the implementation of diffusive models for describing decisional behaviors and the identification of the neuronal areas related to the decisional activity. DM is a process that involves different areas of the brain. These regions include the cortical areas that are supposed to integrate evidence supporting alternative actions and the basal ganglia (BG) that are hypothesized to act as a central switch in gating behavioral requests [9–15]. Neurons in the middle temporal area (MT) are known to encode motion stimulus [13], while the decision process itself occurs in other areas including posterior parietal cortex and prefrontal cortex. Perceptual choice experiments with primates [2, 14] enabled to relate the selective activation of neurons in the lateral intraparietal area (LIP) with the perceptual choice and the response time [15], and this activity would persist throughout a delay between the stimulus and the saccadic movement. This implies that the LIP neurons can respond neither purely to a motor signal, nor simply to sensory input [16]. Rather, LIP neurons are also supposed to contribute to the working memory associated with guiding the eye movement [17]; that is, they would store information about the target location. Neurons in the prefrontal cortex display similar properties during visual motion discrimination tasks [18]. Further studies of human neuroimaging and monkey single-neuron physiology have supported the hypothesis that the parietal and frontal cortices form a system for temporal accruing of data and categorical decision making. These areas would exert executive control on sensory neurons by providing top-down signals that convey information on semantic categorization derived by the stimulus-response association [19, 20].

In natural environments several sensory stimuli produce different alternatives and hence demand the evaluation of different possible responses, that is, a variety of behaviors. In other terms, also a selection question arises [21] whereby the (probability) distribution of the correct response has to take control of the individual's motor plant [22]. The action selection then would resolve a conflict among decisional centers throughout the brain. A central switch that considers the urgency and opportunity of specific response to the stimuli results in an optimal solution in computational terms that is physiologically reliable by taking the basal ganglia (BG) as the neural base for that switch. Accordingly, BG gather input from all over the brain and, by sending tonic inhibition to midbrain and brain stem targets involved in motor actions, block the cortical control over these actions [23–25]. Therefore, the inhibition of the neurons in the output nuclei, caused by BG activity, determines the disinhibition of their targets and the actions would be consequently selected. In other words, BG by acting as a central switch would evaluate the evidence and facilitate the best supported responses [22, 26, 27]. Many studies have reported a significant increase in the firing rate of the neurons of cortical areas representing the alternative choices during DM in visual tasks. The increase of the firing rates then would provide accumulation of evidence (i.e., information) related to the alternatives [1, 2]. The association between the neural firing rates and the DM process is by now an accepted fact and, by the way, some points are worthy to be mentioned. The ramping of the firing rates does not merely anticipate the motor action but would also relate to the target selection. Rather, the rate of growth of the neural activity is proportional to the response times, and so it may predict the decision time. In fact, it triggers the spiking burst of the downstream neurons (in SC and caudate), and the occurrence of their crossing of a defined threshold level marks the decision time. The ramping of the firing rates is also proportional to the prior probabilities of the alternatives and to their probabilities of being rewarded.

**Figure 1:**
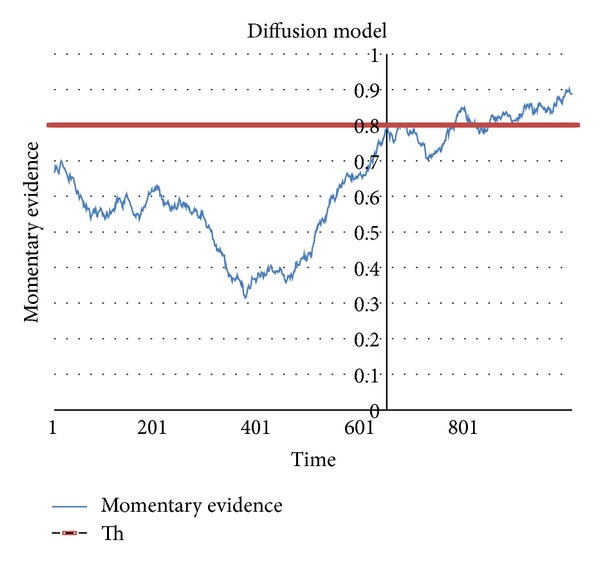
Drift diffusion model. The randomness of the path taken under the influence of noisy stimuli characterizes the diffusion models. A stimulus is represented in a diffusion equation by its influence on the drift rate of a random variable. This random variable, say the difference of evidence corresponding to the alternatives, accumulates the effects of the inputs over time until one of the boundaries is reached. The decision process ends when evidence reaches the threshold, and the time at which it occurs is called response time (RT). Response time (RT) depends on (a) the distance between the boundaries and the starting point, (b) the drift, that is, the rate at which the average (trend) of the random variable changes, and (c) the diffusion, that is, the variability of the path from the trend. These elements characterize the so-called drift diffusion model (DDM). The accumulation of evidence is then driven both by a deterministic component (drift) that is proportional to the stimulus intensity and by a stochastic component of noise (diffusion) that makes the evidence deviate from its own trend. The rationale of DDM is that, since the transmission and codification of the stimuli are inherently noisy, the quality of the feature extraction from such inputs may call for accumulation of a sufficient large sequence of the stimuli to get information [34]. Knowing the threshold level and the RT enables one to take a sight into the mechanism underlying the decision process [12, 88]. We can draw an analogy with a physical system and imagine the decisional process as the state of a “particle” moving within a potential well. Under this point of view, the persistence for relatively long periods of the state variable in the subthreshold area implies that the particle still entangled in the potential well enters an excited state where it remains for an exponentially distributed time interval with a certain decay time *τd*. If the combination of input and noise is sufficiently strong, then the particle is able to jump the barrier, that is, the threshold, and the system returns to an equilibrium state. The dynamics of the particle thus may resolve in a relaxation process [38] characterized by the oscillations between periods of subthreshold “disorder” inside the potential well and short impulses that trigger the system beyond the threshold in the rest state. This physical analogy allows better perception of how the DDM may fit the evolution of the input-output map underlying the neuronal model of the decision making process.

The main purpose of this work was to set the theoretical, neurobiologically sustainable bases for representing the two stages of valuation and choice of DM during Two-Alternative Forced-Choice (TAFC) task in terms of two distinguished layers of neuronal populations performing diffusive dynamics (2LDM), under the assumption that in the DM among alternative options the cortical areas (lateral prefrontal and parietal cortex) integrate the corresponding weighted evidence of the alternatives, whilst the ventromedial prefrontal cortex and the striatum encode the value of different options [28]. The secondary objective was to verify the ability of the 2LDM to account for possible influence that the populations exert over each other. Therefore simulation of time series reproducing the probability of doing motor action (visual targeting) during Two-Alternative Forced-Choice (TAFC) visual task was performed. Power spectrum of the gain functions and synchronization analysis of the instantaneous phases of the activities of the neuronal populations in the two layers of the model suggested activity-dependent modulation of the effective connectivity between the populations. The so-called Two-Alternative Forced-Choice (TAFC) task has often characterized the experimental setting for DM analysis [5, 6, 11]. Bogacz and coauthors [29] evidenced that the TAFC task models typically make three fundamental assumptions: (a) evidence favoring each alternative is integrated over time; (b) the process is subject to random fluctuations; and (c) the decision is made when sufficient evidence has accumulated favoring one alternative over the other. The major issue about the modality of integration of evidence is generally solved in favor of the integration of the difference in evidence, rather than the independent integration of evidence for each alternative. The application of the diffusion models in the study of cognitive processes had been introduced by Ratcliff [5] and since then on they had kept their theoretical soundness in the context of the analysis of decision making under uncertainty [1, 2, 4, 6–11] because it is relatively simple and well characterized [30] and it has been proven to implement the optimal mechanism for TAFC decision making [22, 31]. In applying the diffusion model to the TAFC, it is assumed that the accrual of noisy evidence corresponding to the two alternatives is carried on until their difference reaches a decisional threshold (Figure 1).

It has been shown [5, 31] that, under experiments with human subjects performing TAFC tasks, the drift diffusion model yields accuracy and reaction times (RTs). An advantage from drift diffusion models (DDM) is that, given a level of accuracy, it results in the fastest decision maker, for a fixed decision threshold. The accuracy tends to increase proportionally to the rising of threshold which results in a speed-accuracy tradeoff. This speed-accuracy tradeoff is usually considered a basic parameter for interpreting the results of both behavioral and neurological experiments [12, 15, 32]. The surprising capability of DDM to fit behavioral and neurological data seems to indicate that some decision making processes in the brain are really computed by a similar mechanism that accumulates evidence [33].

However, the canonical diffusion models assume that momentary evidence is accumulated continuously and at* constant rate*, that is, linearly, over the time until a decision threshold is reached [2, 15, 34, 35]. The assumption of linear integration of evidence in human decision making has been recently criticized because it misses the occurrence of refractory periods (“decisional blinks”) during DM, which areknown in psychological literature [36, 37]. On the contrary, the rate of evidence accumulation during DM has been found to fluctuate rhythmically in the delta band as a mechanism of sensory and attentional selection [38–40]. The static linear-nonlinear transfer functions (LN cascades) implemented in the 2LDM for modelling the probability of firing rate of the neuronal populations in response to the stimuli accomplish the hypothesis of nonlinear momentary evidence accumulation. Moreover, in 2LDM a saturating, that is, sigmoidal, activation function is used; therefore, the link between mean population depolarization and expected firing rate (i.e., the input-output map) is parameterized by the slope of the sigmoidal function. Interestingly, the slope is allegedly related to the first- and second-order convolution kernels of the Volterra series, which represent a sufficient specification of population dynamics [41–43]. The driving influence and the activity-dependent modulatory components of the effective connectivity between neuronal populations can therefore be estimated by analyzing the synchronization between the gain functions of the two layers. Specifically, modulatory connections are revealed by asynchronous coupling, whilst driving connections relate to synchronous interactions [44].

Although the evidence accumulation and choice formation are usually described as a one-stage process such that a decision is given as soon as the decisional variable reaches a threshold, it is empirically yet unknown whether decision making is performed in a single neuronal circuit [45]. Merging the two recognized stages of evaluation of alternatives and behavior/motor-action selection into one stage not only renders a model unable to explain overlapping (feature-fusion) phenomenon arising from high-rate succeeding stimuli [46], but seems counterintuitive with respect to the well-established statement that decisions are not unitary events since derivation from two distinct sequential processes [28, 47] as well. Neither would a one-stage diffusion model be able to represent the nonlinear interactions between the cortical and subcortical neuronal populations involved throughout the brain, nor would it properly describe high-conflict over long time scale (>3 s) choices, nor would it yield well distinguished estimation of the times for evaluation and action selection. From a computational point of view, one stage would be a natural outcome only if the activation functions of the computational layers in the multilevel neuronal network were linear, because any multilayer neuronal network, under the condition of linearly separable (i.e., independent) input patterns, might be restricted to a single layer of linear units. However, this assumption is not reasonable in neurobiological context, because it rules out the nonlinear coupling among brain areas, that is, the activity-dependent connections.

The most intriguing two-stage models have been proposed in terms of integrate-and-fire attractor networks [33, 48–50], where the first network evaluates through competitive learning the evidence-biased firing rates of the neurons responding to each of the possible choices and consequently takes a provisional decision in favor of the most valued input. The second network provides final decisions on the base of the confidence in the first level decision, so that changes of the first level decisions are made possible. Positive feedback makes the integrate-and-fire attractor network a nonlinear model and hence it is consistent with the neurophysiology. Strikingly, attractor network exhibits, at local (i.e., cortical) level, nonlinear diffusive dynamics [48], where the biasing input stands for the drift and the stochastic spiking of the neurons provides the diffusion component. Hence, both attractor network model and 2LDM consider decision making inherently a process that involves two levels of computation of nonlinear diffusion dynamics. Also the attractor network model contemplates the role of basal ganglia as driving system of the “global” competition for the action/behavior selection, but in this case through a linear diffusion process. This marks the difference with 2LDM, where the possible implication of BG activity is to be considered in terms of nonlinear diffusion system. As abovementioned, independent, separable input patterns are necessary for linear integration, but this would hinder adaptive mechanisms. Moreover, since the adaptive tuning of the decision threshold is expected to be modulated by reward signals [51, 52], the dopamine-dependent corticostriatal synapses are described as the neurobiological locus for threshold modification [53]. This finding enhances the assumption of nonlinear behavior of BG, whereas spiny projection neurons display* bistable* behavior [54] just because bistability calls for nonlinearity, feedback, and hysteresis, which are conditions consistent with the implementation of reinforcement learning in BG.

The paper is organized as follows.

Section 2 is about the neuronal populations codes and the relationship between interpulse intervals and response times. The last part of the section is dedicated to the description of the two-layered diffusion model (2LDM).

Section 3 presents the results of synchronization analysis between the instantaneous phases of the activities of the two neuronal populations and the power spectrum of the gain functions from the application of the 2LDM over simulated data that were obtained by resampling time series of the probability of visual targeting (likelihood) recorded during a Two-Alternative Forced-Choice (TAFC) visual task.

Section 4 summarizes and discusses the main results and adds some comments about computational and neurobiological implications or potential developments of the 2LDM.

The appendix deals with statistical theory on distances between features.

## 2. Two-Layered Diffusion Model (2LDM)

### 2.1. Population Code

As long as the cells in a neural population have similar response properties, that is, acting in a statistically similar way [55], then the brain collects and organizes information from patterns of activity involving populations of neurons [56, 57]. In the work of Sanger [55] it is also described that the input-output (stimulus-response) map stems from the modulation of information; that is, calculation on values that are represented by population codes (encoding) and feature extraction about the input stimuli (decoding), in the brain, may be seen as relations between different population codes that provide internal representations of the input-output map. In this perspective, then, computation in the brain relies on commutations from one internal representation to another. By assuming that populations of neurons regulate the responses to stimuli, we can consider the effect of the accumulation of activity from a combination of two neural populations *P*_1_ and *P*_2_. The gathering and processing of information during the experiments then would elicit spike-trains from those cells of *P*_*k*_  (*k* = 1,2) within the interval [0, *T*]. If we count the number of spikes emitted up to a time *t*  (*t* ≤ *T*), we obtain the variables *N*_*k*_(*t*) that represent the sequence of evidence accumulation. Over the time, the occurrence of noise makes *N*_*k*_(*t*) stochastic variables, and so the process of accumulation of spikes from the neural population traces a random pattern that is expected to end as it encounters a bound (*θ*_*k*_) at a finite time *τ* [12]. The two processes {*N*_*k*_(*t*), *t* ≤ *T*} by which the neural system learns the input stimuli *I*, thus, determine the behavior of the decisional variable. This learning activity then would give rise to a first level of codification through *P*_1_ that provides the elaboration and probabilistic valuation of the input *I*. In fact, we can imagine a binary code where the “1 s” corresponds to *N*_1_*θ*1__, that is, to the times the bound *θ*_1_ is trespassed. Afterwards, the codification from *P*_1_ is “translated” into the population *P*_2_ by the variable *N*_2_*θ*2__. This provides another binary code (0,1) based on the overtaking of the threshold *θ*_2_, which ultimately drives the eye movements during the computational task. Calculation on values that are represented by population codes and feature extraction about the input stimuli in the brain may be seen as relations between different population codes that provide internal representations of the input-output map. Hence, we can “translate” the likelihood of *y*∣*x* into a pulsed binary code *y*_*δ*_, say the *δ*-code, where *δ* is a nonlinear transform of *θ*_2_ such that *δ* = *g*_2_(*θ*_2_) and *y*_*δ*_ at time *t* assume the value “1” (pulse) if the likelihood (*y*∣*x*) > *δ*, or the value “0” (no-pulse) otherwise.

### 2.2. Interpulse Intervals and Response Times

**Figure 2:**
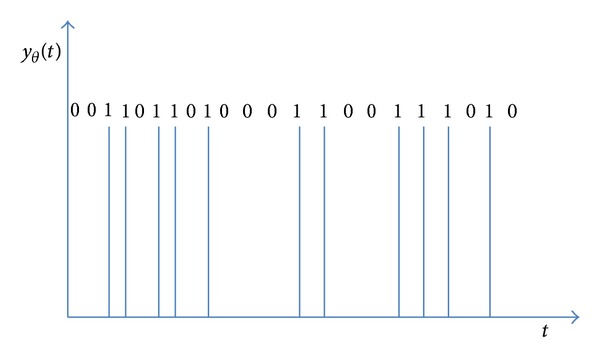
Example of binary encoding of information. The threshold value *θ*_2_ allows reading of the variable *y* as a binary code where the 1 s pulses occur when (*y*∣*x*) = *g*(*N*_2_) > *g*_2_(*θ*_2_). The lengths of the sequences of zeros provide the interpulse-intervals (IPI).

After the signal (*y*∣*x*) has been reformulated on the base of the *δ*-code (Figure 2), we obtain a string of symbols (0,1) and the lengths of the sequences of the zeros provide the holding times, that is, the empirical interpulse intervals, IPI(*δ*); that is, the variable *y* recoded according to *δ* results in periods of subthreshold location that are broken out by sequences of impulses. Analogously, from the recoded *N*_2_*θ*2__ we obtain the corresponding empirical holding times IPI(*θ*_2_) given the transform *g*_1_(*N*_1_*θ*1__) = *N*_2_*θ*2__. Thus we can imagine a functional chain among the bounds *θ*_1_, *θ*_2_, and  *δ* scaled by some opportune nonlinear transfer functions *g*_1_, *g*_2_ (without loss of generality, we set *g*_1_ = *g*_2_ = *g*). Let us assume that {*N*_*k*_(*t*)}  (*k* = 1,2) behaves a renewal process. The expected value and the variance of a renewal process may be obtained from the observed IPI data. In fact, for large *t*, the variable *N*_*k*_(*t*) is normally distributed with mean *t*/*μ* and variance *t* · *σ* ^2^/*μ* ^3^, where *μ* and *σ* are the mean and the standard deviation of the corresponding IPI sequence, respectively [58, 59]. Therefore, the time series *N*_*k*_(*t*) can be reconstructed by averaging out over the neural population a Gaussian random variable with mean *t*/*μ* and variance *t* · *σ* ^2^/*μ* ^3^.

The importance of IPI arises from the hypothesis that the information transferred within the nervous system is usually encoded also by the timing of spikes [60–62]. (Since we are dealing ultimately with the threshold-dependent variable *y*_*δ*_, the random variable *T*_*θ*_ = inf⁡{*t* ≥ 0∣*N*_*k*_(*t*) ≥ *θ*_*k*_} is implicitly involved, where *N*_*k*_ is the scalar diffusion process *N*_*k*_ = {*N*_*k*_(*t*), *t* ≥ 0} that describes the evolution of the potential, that is, of the evidence, between two consecutive neuronal firings. *T*_*θ*_ then is the theoretical counterpart of the IPI.) Thus, by studying the properties of the set of the times *t* that correspond to the crossings of the threshold we both realize the relationship between the impulse rate of the variables *y*_*δ*_ and IPI and solve the so-called first passage time problem [63] and hence the response time problem as well. We can then consider the IPI as the expression of the response time of the process through the threshold. Given this association, it becomes natural to compare the theoretical distribution of the response times to the observed distribution of the IPI. An impulse of the variable *y*_*δ*_ is elicited any time the process *g*(*N*_2_) crosses the threshold *g*(*θ*_2_) and then *g*(*N*_2_) starts again according to a renewal process. (This assumption is necessary to identify the time series of successive pulses times as a sample extracted from a population with the same distribution of the random variable *T*_*θ*_ [64].) The question then is how to model the distribution of the response times. We hypothesized the ex-Wald distribution of the response times [65] so that the cumulative distribution function of the response time variable is given by

(1) H(t ∣ v,s,θ,γ)=F(t ∣ v,s,θ)−F(t ∣ k,s,α)·e−γ·t+((θ·(v−k))/s2),

where *s* is the diffusion parameter (i.e., noise of the process), $k=\sqrt{v^{2}-2\cdot \gamma \cdot s^{2}}$, *v* and *θ* are the drift and the threshold of the diffusion process, and *γ*  (>0) is the rate parameter of the exponential distribution. *F* is the cumulative distribution function of the Wald distributed component of the response time variable and Φ is the standard Gaussian distribution:

(2) F(t ∣ v,s,θ)=Φ(v·t−θs·t)+e(2·θ·v)/s2·Φ(−v·t+θs·t).

The estimation of the parameters (*s*, *θ*, *v*, *γ*) of the response time distribution involves a backward procedure. Firstly, the variable *y*_*δ*_ must be determined for an initial value of the threshold *δ* = *δ*_0_ so as to obtain the distribution of the corresponding IPI(*δ*_0_). Secondly, the best combination of the parameters (*s*_2_, *θ*_2_, *v*_2_, *γ*_2_) for the RT distribution of *N*_2_ will be assigned as the one which minimizes some error function, say the root mean square error (RMSE) of the difference between their corresponding distribution of RT and the observed IPI(*δ*_0_). (Indeed the RMSE is a quadratic function of the errors and is optimal when the residuals are distributed as normal random variables. In that occurrence the RMSE is a convex surface. On the contrary, in presence of heavy-tailed distributions of the residuals, the RMSE becomes suboptimal, and it had better use other criteria, e.g., based on entropy measures. However, in front of the computational complexity involved in the inverse method for deriving the parameters of the diffusion models, the RMSE may turn out to be economical.) Lastly, the parameters (*s*_1_, *θ*_1_, *v*_1_, *γ*_1_) for the RT distribution of the variable *N*_1_ can be estimated analogously by comparison to the interpulse-interval distribution IPI(*θ*_2_). Of course the final result of the sequential procedure is *δ*_0_-dependent; that is, the particular initial value of *δ*_0_ may affect the estimated vector of optimal parameters. Therefore, the question is how to initialize *δ*_0_. The assignation to the average of the likelihood, that is, *δ*_0_ = *E*(*y*∣*x*), suggests an interesting interpretation under the information theory perspective. In fact, we may expect that the function *y*_*δ*_ = *g*(*N*_2_*θ*2__) should be learned so as to maximize the mutual information between *y* and *N* subject to noise effect. This is the so-called infomax principle, by which the process of stimuli-learning gives rise to optimization algorithms [66]. If the noise that affects the system is Gaussian and independent of the input *N*_2_, then the mutual information between *y* and *N*_2_ resolves in the difference between the entropy of the output *y* and the entropy of the noise [60]. It implies that, to improve the information transmission, the entropy of the signal *y* must be maximized. Therefore, the value of the function *y*_*δ*_ = *g*(*N*_2_*θ*2__), which corresponds to the maximal entropy of the binary signal *y*_*δ*_, is expected to be very close to the one that corresponds to the average *E*(*y*∣*x*). To recover the mapping from stimulus to impulse rate we can apply a nonlinear transformation *g* of a convolution of the stimuli *N*_1_, *N*_2_. By assuming the logistic function for the nonlinear transforming function we can write *g* = 1/(1 + *e* ^−*X*(*t*)^), where *X*(*t*) = (*C*∗*N*)(*t*) is the convolution of the stimuli *N* (i.e., *N*_1_ or *N*_2_) with an opportune function *C* that is obtained in two stages. In the first stage the transfer function estimate *T*_*Nz*_ is computed for the input signal *N* and the binary output signal *z*, where *z* is the variable representing the probability of impulse rates, that is, *y*_*δ*_ if *N* = *N*_2_ or *N*_2_*θ*2__ if *N* = *N*_1_. The relationship between *N* and *z* is shaped by the static (i.e., time invariant) transfer function *T*_*Nz*_, that is, the ratio of the cross power spectral density of *N* and *z* over the power spectral density of *N*. In the second stage, the inverse discrete Fourier transform of *T*_*Nz*_ is computed. Since the inputs *N* are generated from Gaussian process, then *X*(*t*) = (*C*∗*N*)(*t*) is Gaussian too. According to the theorem of Bussgang, the cross-correlation between *z* and *X* scales the autocovariance function of *X* by a value alpha = *E*[*X* · *g*(*X*)]/var(*X*); therefore we can correct *C* with *C*′ = *C*/alpha. Next, the convolution of vectors *N* and *C*′ forms the argument of the logistic transfer function *g*. This procedure yields a static linear-nonlinear model for the probability of firing rate of the neuronal populations in response to the stimuli and the variable *z* is then expressed by *z*(*t*) = *g*((*C*′∗*N*)(*t*)) + *r*(*t*), where *r*(*t*) is the noise term [67].

### 2.3. Structure of the Model

**Figure 3:**
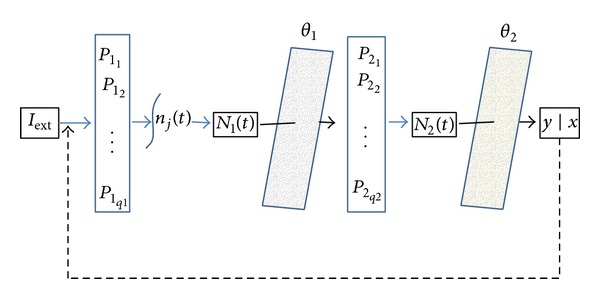
The two-layered diffusion model (2LDM) for decision making. Both stages (valuation and choice) are affected by noise. In the valuation stage the critical threshold indicates the firing rate of the neuronal populations involved, to which would correspond the expected reward. The outputs of this stage then are the differences between the responses of observed neuronal activity at the stimuli provided by the alternatives and the target. These measurements enter the next stage, where the decision is taken so as to optimize some utility criterion (reward). Hence, the attainment of the threshold in the decision stage indicates the preferred alternative. Feedback information flows from the decision stage in order to elicit the adaptation of the boundary in the valuation layer. In this way, a mechanism of reinforcement determines the competition between the alternatives and the valuation is biased to the most probable rewarded one.

Let us consider the input-output map between input *I* and the final state of decisional variable *x*. Data inflow at time *t*; *I*_*t*_ proceeds from external input *I*_ext_ and recurrent output obtained at time *t* − 1  (*y*∣*x*)_*t*−1_. This relation, which implies relatively complex computational paradigms, is mediated by populations of neurons *P*_1_ and *P*_2_ in different areas in the brain. Cells (*P*_1_1__,…, *P*_1_*q*1__) of neuron population *P*_1_, activated by input *I*, respond according to a tuning curve *s*_*j*_(*I*)  (*j* = 1,…, *q*) and generate the time series of spikes *n*_*j*_(*t*). Variable *N*_1_(*t*) counts the spikes until the threshold *θ*_1_ is reached. This event affects the observable variable *y* and thus the final decisional state *x* through a second neuron population *P*_2_. The firing of *P*_2_ neurons is integrated in variable *N*_2_(*t*) that exceeds threshold *θ*_2_ and ultimately drives the path of the variable *y*. The state of *y* at any time *t* holds the whole information-set available up to *t*, including the implicit reward corresponding to the *y* state at that time. By aiming at the maximization of the reward, the system would give rise to gap evaluation and error reduction that ultimately involves a feedback circuitry. In this way, the information backpropagates from the decision stage to the valuation stage in order to elicit the adaptation of the threshold *θ* in the valuation stage. This mechanism of reinforcement triggers the competition between the alternatives and the valuation is ultimately addressed to the most probable rewarded one (Figure 3).

## 3. Simulation

### 3.1. Methods

In order to test the ability of the model to detect effective interactions between the neuronal populations, simulation of the 2LDM was carried on by resampling time series of conditional probabilities from a previous experiment of eye tracking. Nine subjects had been asked to look at two abstract images displayed on a screen for 5 seconds (s) at randomly assigned locations (left or right side). Each subject performed ten trials. The two images were balanced by extension and by photometrical characteristics (color, luminance, and contrast). Eye movements had been recorded during the period of 5 s (sampling frequency 1/50 ms) and at the end of that time subjects declared which of the images was their preferred one. The likelihood, that is, the probability of visual targeting towards one of two images conditional to the final chosen stimulus, was then calculated over the total 90 choices. One hundred surrogates of this likelihood time series were obtained by using the iAAFT technique (iterated amplitude adjusted Fourier transform) [68], so preserving the marginal distribution and power spectrum of the original signal. Next, Gaussian noise proportional to the standard deviation of the original likelihood was added at thirty randomly selected points in each surrogate series. Run test was applied to the resulting modified iAAFT surrogates, and were retained only the ones for which the null hypothesis of mutually independence of the elements in the sequence was rejected. This procedure guaranteed the generation of forty realistic-structured data vectors to which the 2LDM was implemented. Paired *t*-test for comparing the rates of populations' activity variables *N*_1_ and *N*_2_ was done. Power spectrum of the gain functions *g*((*C*′∗*N*)(*t*)) calculated for the two layers was reported. Hilbert-transforms of the average rates of populations' activity variables *N*_1_ and *N*_2_ were produced so as to derive their instantaneous phases (*φ*_1_, *φ*_2_) [69]. The* correntropy* coefficient (*η*), which is a measure of correlation in the reproducing kernel Hilbert space (RKHS) ranging over [−1, + 1], proper for nonlinear relationship [70], operated as coefficient of phase locking (i.e., synchronization) between the phases *φ*_1_ and *φ*_2_. Correntropy measures were calculated dynamically, that is, in running windows (of depth = 6 data points), over the phase signals.

To use the phase locking indices in a meaningful way, we need to know their distribution under the null hypothesis of independent pairs of oscillatory activity. Only values that depart significantly from what would be expected for independent oscillators can be considered as revealing the presence of synchronization. The distribution of the index, computed for pairs drawn randomly from the surrogate ensembles, can be considered as an approximation of the distribution under the null hypothesis [71]. Therefore, the iAAFT surrogates of both average rates of populations' activity variables *N*_1_ and *N*_2_ were Hilbert-transformed and the resultant instantaneous phases (*φ*_sur1_, *φ*_sur2_) attained, and the time series of correntropy values (*η*_sur_) over running windows (of the same size as before) between *φ*_sur1_ and *φ*_sur2_ was obtained.

To test the null hypothesis that the mean of the distances between features (*η* and *η*_sur_) is zero, the Weibull-like distribution of the variable *D* = *η* − *η*_sur_ was considered (see the appendix).

### 3.2. Results

**Figure 4:**
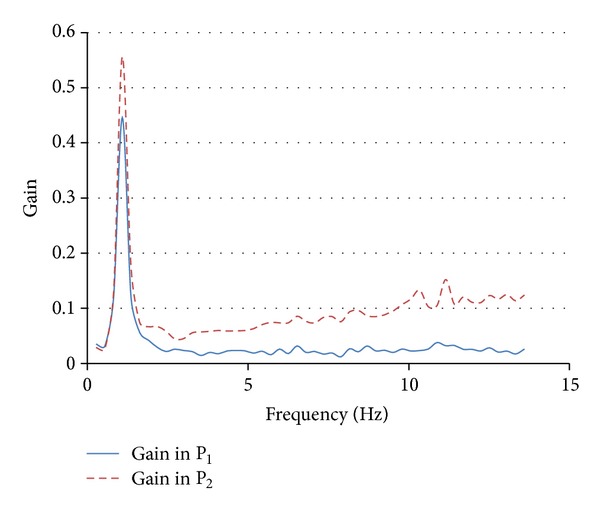
Gain functions. In the plot are displayed the gain functions relative to the neuronal populations of the two layers. Both showed prominent rhythmic activity in the delta band. Increased oscillations up to beta band characterized population *P*_2_.

**Figure 5:**
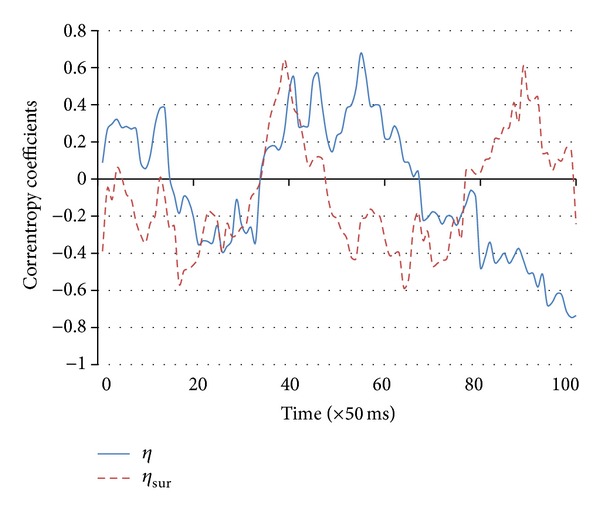
Time course of the correntropy coefficient between the phase signals in *P*_1_ and *P*_2_, (*η*), and between the surrogate phases (*η*_sur_). Correntropy is a measure of nonlinear correlation that is obtained by the projection of the original vectors onto the reproducing kernel Hilbert space. The plot displays the time course of correntropy coefficients (*η* and *η*_sur_) between phase signals *φ*_1_ and *φ*_2_ and between the corresponding surrogate phase signals (*φ*_sur1_ and *φ*_sur2_). Zero values correspond to independence between the signals.

**Figure 6:**
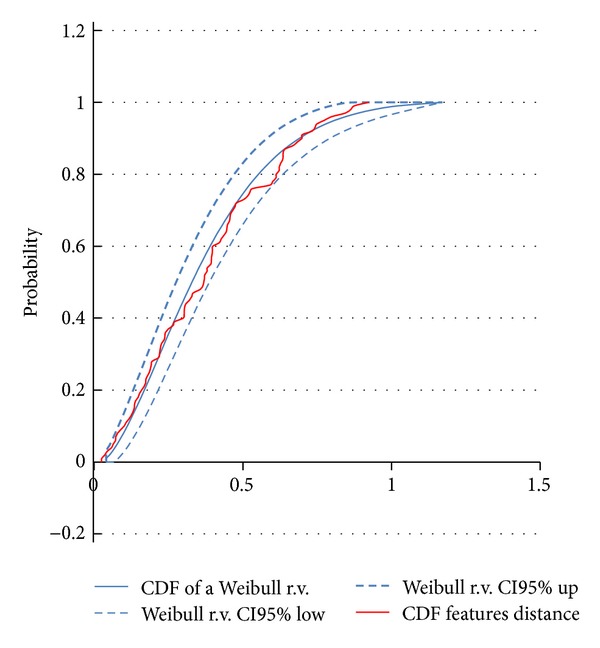
Cumulative distribution function of distance between *η* and *η*_sur_. The cumulative distribution function of the variable representing the difference between the correntropy coefficients *η* and *η*_sur_ is distributed as a Weibull-like random variable (with parameters *a* = 0.3752 and *b* = 1.5661).

**Figure 7:**
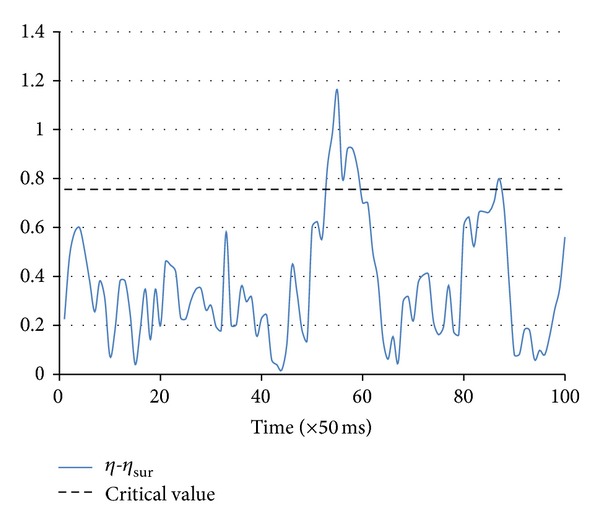
Distance of the correntropy coefficients measured for the phase signals and their surrogates. Synchronized interaction between the two neuronal populations was determined in correspondence with the values of the correntropy distance vector above the critical value (0.756), which was calculated according to the distribution of a Weibull random variable with parameters *a* = 0.3752 and *b* = 1.5661 at the significance level of 5%. We observed a prominent asynchronous interaction.

The resampled time series of the likelihood of visual targeting at the final selected image ranged over [0.3827 : 0.7427], with mean = 0.5853 and SD = 0.095. The original likelihood data series had mean = 0.6630 and SD = 0.0951. A paired-samples *t*-test was conducted to compare the rates of populations' activity variables  *N*_1_ and *N*_2_. There was a significant difference between the rates of *N*_1_ (mean = 0.2775, SD = 0.001) and the rates of *N*_2_ (mean = 0.1891, SD = 0.0009); *t*(99) = 649.85; *P* = 0.00001. Power spectrum of the gain function in *P*_2_ showed higher components than in *P*_1_ up to the (lower bound of) beta-band (Figure 4). Level of synchronization between the instantaneous phases (*φ*_1_, *φ*_2_) calculated by the Hilbert-transform of the rates of populations' activity variables  *N*_1_ and *N*_2_ was determined in terms of correntropy coefficients *η* (Figure 5). Departures from zero values indicate phase locking. To test the null hypothesis of asynchronous state, the vector of correntropies *η*_sur_ between the surrogate instantaneous phases (*φ*_sur1_, *φ*_sur2_) was considered as representative of the null hypothesis. The distance between *η* and *η*_sur_ was expected to be distributed as a Weibull random variable (see the appendix and Figure 6) with shape and scale parameters *a* = 0.3752 and *b* = 1.5661. According to the Weibull-like distribution, we found that the test statistic, mean (distance)/S.E.(distance) = 15.323, was significantly different from zero (*P* = 0.00012). Values in the distance feature vector greater than the critical value = 0.756 (for the significance level of 5%) revealed the times of synchronization occurrence (Figure 7). Synchronized activities of the neuronal population were concentrated in the time interval between *t* = 54 and *t* = 59 and peaked also at *t* = 86.

## 4. Conclusions

The model presented in this study assumes that the trajectories of an observable variable (*y*) induced by the TAFC decision making task are conditional to the final state (*x*), and so they trace the information processing. Under this hypothesis, the possible association between the formation of a decision, as determined by the *y* path, and the final state of the decisional process can be investigated by considering that populations of neurons determine neuronal responses to stimuli (Figure 3). More specifically, here it is hypothesized that the series of the likelihood (*y*∣*x*) are generated by sequential activation of two neuronal populations *P*_1_ and *P*_2_ and that the decisional process is the effect of accumulation of activity by a pool of neuron populations. This would engender diffusive dynamics of the accumulated evidence. Thus, the proposed model, 2LDM, is, to a certain extent, an implementation of the two-stage circuitry of valuation and decision which is computationally reliable in terms of both neurobiology and Bayesian theory [72, 73]. From this perspective, likelihood ultimately relies on commutations from one internal representation to another, according to their diffusive processes of activation.

There is a theoretical linkage between 2LDM and the well-recognized integrate-and-fire attractor network model [33, 48–50] since both models rely on nonlinear diffusive dynamics. Major difference rests in the expected dynamics of the basal ganglia involved during the decision making process, which we considered driven by nonlinear patterns rather than linear patterns. Furthermore, the characterization of the input-output map in terms of the infomax principle makes, ultimately, the 2LDM an entropy-thresholding algorithm where the model's parameters (threshold, diffusion noise, and drift) should be tuned to maximize the mutual information between the representations they engender and the inputs that feed the layers. This is consistent with the Q-learning adaptation, since learning the “best” action on the two thresholds to maximize the cumulative entropy is equivalent to learning the optimal behavior which maximizes the reward [74, 75]. Nonlinearity in the 2LDM is given by static linear-nonlinear functions that express the gain of the input-output map, so overcoming the theoretical weakness inherent in the canonical diffusion models which assume that momentary evidence is accumulated continuously and at constant rate, that is, linearly, until a decision threshold is reached. This way to model nonlinear dynamics is not a novelty in neuroscience because it fits for Volterra series representation which, through the first- and second-order kernels, estimates the driving and modulatory influence that one population exerts on the other. The slope of sigmoidal transfer function yields information about the effective connectivity between the neuronal populations, because it is a proxy of the Volterra kernels [76].

Simulation was used to test the ability of 2LDM to represent interactions between the neuronal populations on reliable time series and did not aim at investigating the underlying cognitive process. Synchronous interaction was present within a restricted median time interval, where, supposedly, the dynamics of the two neuronal populations were mutually reinforcing [44]. Instead, asynchronous interaction was prominent. This kind of finding is expected for modulatory (i.e., top-down) connections rather than for driving influence. Neurobiological consistency of the results was found also in terms of the power spectrum of the gain functions, which showed rhythmic oscillations in the low-frequency bands (from delta- to beta-bands). The spectral content of neuronal activity in the circuits of valuation and choice may yield information about the mechanisms underlying the DM [77]. In fact, neuronal oscillations are associated with reverberating activity at local and large scale [78] and reverberation would elicit prolonged accumulation of evidence during decision making [79]. Delta-band oscillations in cortical areas have been associated with attention [80], while the occurrence of synchronization in the delta-band is reported to be widespread and modulated by the different decision alternatives and context specific [81]. Theta-band oscillations are expected to operate in many cognitive functions including memory and DM [82, 83]. The striatum oscillations in theta frequency range are prevalent but activity in lower band is also observed [84]. In a study of DM [85], oscillations in alpha and beta frequency bands had been found synchronized with the phase of delta and theta oscillations (*phase-amplitude coupling*) in medial frontal cortex. This synchronization might reflect a mechanism of feedback valence coding in the medial frontal cortex. Beta-band activity has been linked to reverberation, which is a possible mechanism for memory consolidation and accumulation of evidence [86], as well as to computational operating in DM rather than neuronal representation of the sensory evidence [87]. Our finding of increased beta activity (although in the lower bound of the beta frequency range) in the second neuronal population, which would perform the selection of the optimal alternative, seems consistent with this latter perspective.

Improvement in the optimization of the 2LDM parameters is expected by considering other error functions instead of RMSE if the distribution of the residuals is not Gaussian and is heavy-tailed such that it exhibits large skewness and kurtosis. A challenging task would be the implementation of further layers for studying the subcircuits possibly involved in the valuation or choice stage of DM (e.g., the direct and indirect pathways in BG). Finally, the application of 2LDM to specific cognitive experimental task would yield information about how the speed-and-accuracy performance may vary on the base of some psychometric or behavioral smoothing parameter.

## Appendix

### Statistics of Distances between Features

To measure the similarity between two feature vectors, many distance measures have been proposed [89, 90]. A common metric class of measures is the *L*_*p*_-norm. The distance from one reference vector **s** to another feature vector **t** can be formalized as

(A.1) $$\begin{array}{c} D={\left(\underset{i}{\sum }{\left|s_{i}-t_{i}\right|}^{p}\right)}^{1/p}=\underset{i}{\sum }X_{i}^{p}. \end{array}$$

In order to derive the distribution of the variable *D* we can refer to the following.

Lemma A.1 A.1. For nonidentical and correlated random variables *X*_*i*_, their sum is distributed according to the generalized extreme value distribution (Gumbel, Frechét, and Weibull).

Lemma A.2 A.2. If in Lemma A.1 the variables *X*_*i*_ are upper bounded, the sum of variables is Weibull-distributed.

Theorem A.3 A.3. For nonidentical, correlated, and upper bounded variables *X*_*i*_, the random variable *Y*, expressed by their sum, adheres to the Weibull distribution.

Corollary A.4 A.4. For finite length feature vectors with nonidentical, correlated, and upper bounded values, the *L*_*p*_-distances for limited *p*, from one reference feature vector to other feature vectors, adhere to the Weibull distribution.
